# Self‐Reported Liver Disease and the Burden of Erythropoietic Protoporphyria

**DOI:** 10.1002/jmd2.70015

**Published:** 2025-04-20

**Authors:** Hetanshi Naik, Kristen Wheeden, Hilary H. Colwell, Susan D. Mathias, Chelsea Norregaard, Melanie Chin, William Savage, Manisha Balwani

**Affiliations:** ^1^ Department of Genetics Stanford University Stanford USA; ^2^ United Porphyrias Association Bethesda USA; ^3^ Health Outcomes Solutions Palm Beach Gardens USA; ^4^ Disc Medicine Watertown USA; ^5^ Department of Genetics and Genomic Sciences Icahn School of Medicine at Mount Sinai New York USA

**Keywords:** erythropoietic protoporphyria (EPP), healthcare utilization, health‐related quality of life, liver disease, patient‐reported outcomes, phototoxicity

## Abstract

Erythropoietic protoporphyria (EPP) and X‐linked protoporphyria are metabolic disorders that cause skin phototoxicity and potential liver damage. We compared symptoms and impacts of phototoxic reactions, health‐related quality of life, and healthcare utilization (HCU) between individuals with and without self‐reported liver disease (elevated liver enzymes or liver fibrosis) using an online questionnaire containing validated patient‐reported outcome measures and original items. Among 164 participants, 15.2% self‐reported liver disease. Sixty‐four percent of those with liver disease rated their general health “much worse” than those without EPP, versus 35% of those without liver disease. Those with liver disease had a higher frequency of prodromal symptoms and more frequently reported that their most recent phototoxic reaction impacted their ability to perform daily activities (76% versus 55% for those without liver disease) and resulted in difficulty in doing chores (88% versus 60%) or going for a walk, run, or bike ride (84% versus 60%). A higher percentage of those with liver disease reported feeling anxious (92% versus 78%), isolated (100% versus 80%), and lonely (96% versus 72%) than those without liver disease. The mean number of hours missed from work and school in the past month was higher for those with liver disease (work: 10.9 h; school: 7.7 h) than those without liver disease (3.6 h for both). EPP‐related HCU in the previous 12 months was higher for those with liver disease than those without liver disease, including more physician visits (mean of 16.5 versus 6.0) and emergency room visits (mean of 9.0 versus 1.9).

1

**Summary**

- Among individuals with erythropoietic protoporphyria, those with self‐reported liver disease reported a higher symptom burden, more interference with daily activities and work, worse health‐related quality of life, and higher healthcare utilization than those without liver disease.

## 2 Introduction

The most serious risk associated with erythropoietic protoporphyria (EPP) is liver disease [1]. EPP (and X‐linked protoporphyria, also referred to as EPP here) is a rare, metabolic condition where protoporphyrin IX (PPIX) is overproduced and accumulates in erythrocytes, plasma, skin, and the liver [1, 2, 3]. In EPP, PPIX progressively accumulates in the liver, and high concentrations of it can result in crystallization, forming biliary stones and causing architectural damage within the hepatobiliary system. Approximately 20% of patients with EPP develop gallstones, with up to 5% of patients progressing to liver failure [2, 4, 5, 6]. EPP imposes a substantial burden on individuals and negatively impacts health‐related quality of life (HRQoL) [7, 8, 9, 10]. However, data describing the experiences and healthcare utilization (HCU) of those with liver disease are lacking. Therefore, we explored responses from a cross‐sectional, online questionnaire of individuals with EPP who self‐reported liver disease, as defined by elevated liver enzymes or liver fibrosis, and compared them with responses from those who did not report liver disease.

## 3 Methods

### 3.1 Participants

Participants for this study were identified with assistance from the United Porphyrias Association, a patient advocacy group. To be eligible, individuals were required to be ≥ 12 years of age with a diagnosis of EPP and to reside in the US or Canada. Results presented herein reflect a subgroup analysis of a larger study of adults and adolescents with EPP. This subgroup analysis from the EPP Life Impact and Genetic Health Trajectory (LIGHT) study is limited to adult participants ≥ 18 years of age, because only 1 adolescent self‐reported liver disease. Participants provided informed consent, and the study was approved by an independent scientific review committee, WCG IRB. Participants were remunerated for their time.

### 3.2 Questionnaire and Data Collection

An online questionnaire was used to collect information from participants regarding their experiences with EPP. Specifically, participants were asked about their general health, the frequency and severity of prodromal symptoms and phototoxic reactions, and the associated impacts on physical functioning, emotional well‐being, work/school loss/productivity, and HCU (hospitalizations and emergency room [ER] visits).

The questionnaire included items from existing, validated patient‐reported outcome (PRO) measures developed based on qualitative research with individuals with EPP and original PRO items developed by the authors, specifically for this study. This included select questions from the EPP Impact Questionnaire (EPIQ) [11] and items assessing prodromal symptoms and pain from EPP (Sunlight Exposure Diary) [12], among others. Participants also provided demographic and clinical information. Liver disease was defined as elevated liver enzymes or liver fibrosis, which was self‐reported by participants and not confirmed by clinicians.

This was an exploratory study with no pre‐specified hypotheses. Therefore, no formal hypothesis testing was conducted. Comparisons between those with and without liver disease are descriptive.

## 4 Results

### 4.1 Respondent Characteristics and General Health

Among a total of 164 adult participants, 25 (15.2%) self‐reported liver disease and 139 did not. Results can be compared to those from a study of 114 adults with EPP in which liver disease was confirmed by labs, abdominal ultrasonography, and transient elastography. In this comparison study, elevated liver enzymes were found in 6%, liver steatosis was reported in 29%, and significant fibrosis as assessed with liver stiffness measurements was present in 10% of patients. It should also be noted that the prevalence of liver disease in this study was similar to the prevalence of liver disease in the Dutch general population [13].

Those who reported liver disease had similar demographic characteristics to those without liver disease: the majority of individuals within both groups were female, White, and non‐Hispanic. However, those with liver disease reported a higher prevalence of several comorbid conditions, including anemia (76% vs. 55%), gallstones (44% vs. 28%), depression (56% vs. 22%), and anxiety (52% vs. 22%) (Table 1). Among those with liver disease, the mean (standard deviation [SD]) time since receiving a liver disease diagnosis was 8.6 (8.0) years.

**TABLE 1:** Demographic and clinical characteristics.

	Full sample (*N* = 164)	No liver disease (*N* = 139)	Liver disease (*N* = 25)
Age in years, mean ± SD (range)	45 ± 15 (18–82)	45 ± 15 (18–82)	42 ± 14 (18–69)
Gender, *n* (%)			
Male	63 (38%)	54 (39%)	9 (36%)
Female	98 (60%)	83 (60%)	15 (60%)
Non‐binary	3 (2%)	2 (1%)	1 (4%)
Ethnicity, *n* (%)			
Hispanic or Latino	10 (6%)	7 (5%)	3 (12%)
Not Hispanic or Latino	151 (92%)	130 (94%)	21 (84%)
Not sure	3 (2%)	2 (1%)	1 (4%)
Race, *n* (%)			
White	158 (96%)	135 (97%)	23 (92%)
Black or African American	3 (2%)	2 (1%)	1 (4%)
American Indian/Alaska Native	2 (1%)	1 (1%)	1 (4%)
Asian	2 (1%)	2 (1%)	0 (0%)
Native Hawaiian/Pacific Islander	2 (1%)	0 (0%)	2 (8%)
Other	4 (2%)	4 (3%)	0 (0%)
Work/school status, *n* (%)			
Work full time for pay	88 (54%)	77 (55%)	11 (44%)
Work part time for pay	22 (13%)	18 (13%)	4 (16%)
Do not work due to EPP	9 (6%)	7 (5%)	2 (8%)
Do not work, unrelated to EPP	14 (9%)	12 (9%)	2 (8%)
Go to school part time	8 (5%)	6 (4%)	2 (8%)
Go to school full time	12 (7%)	8 (6%)	4 (16%)
Other*	40 (24%)	35 (25%)	5 (20%)
Age at diagnosis, mean ± SD	15 ± 13	16 ± 12	14 ± 13
Median (25–75 percentiles)	12 (6–23)	12 (6–22)	8 (4–25)
Time to receive diagnosis, *n* (%)			
< = 1 year	31 (19%)	26 (19%)	5 (20%)
> 1 to 4 years	30 (18%)	25 (18%)	5 (20%)
5 to 9 years	34 (21%)	27 (19%)	7 (28%)
≥ 10 years	69 (42%)	61 (44%)	8 (32%)
Other comorbid conditions, *n* (%)			
Liver disease	25 (15%)	0 (0%)	25 (100%)
Gallstones	50 (31%)	39 (28%)	11 (44%)
Liver disease or gallstones	64 (39%)	39 (28%)	25 (100%)
Anemia	95 (58%)	76 (55%)	19 (76%)
Vitamin D deficiency	97 (59%)	82 (59%)	15 (60%)
Depression	44 (27%)	30 (22%)	14 (56%)
Anxiety	44 (27%)	31 (22%)	13 (52%)
Osteopenia	15 (9%)	14 (10%)	1 (4%)
Other	20 (12%)	18 (13%)	2 (8%)
None	21 (13%)	21 (15%)	0 (0%)

* Includes combination of working for pay and attending school.

Sixty‐four percent of those with liver disease rated their general health “much worse” than those without EPP, compared with 35% of those without liver disease (Table 2).

**TABLE 2:** Self‐Rated general health and prodromal symptoms, by self‐reported liver disease.

	Full sample (*N* = 164)	No liver disease (*N* = 139)	Liver disease (*N* = 25)
Self‐rated general health, *n* (%)			
Excellent	18 (11%)	18 (13%)	0 (0%)
Very good	53 (32%)	49 (35%)	4 (16%)
Good	60 (37%)	53 (38%)	7 (28%)
Fair	21 (13%)	13 (9%)	8 (32%)
Poor	12 (7%)	6 (4%)	6 (24%)
Self‐rated general health versus those without EPP, *n* (%)			
Much worse	64 (39%)	48 (35%)	16 (64%)
A little worse	72 (44%)	66 (48%)	6 (24%)
The same	21 (13%)	19 (14%)	2 (8%)
A little better	5 (3%)	4 (3%)	1 (4%)
Much better	2 (1%)	2 (1%)	0 (0%)
Time in sunlight before having prodromal symptoms, *n* (%)			
≤ 10 min	80 (49%)	68 (49%)	12 (48%)
> 10 to ≤ 30 min	57 (35%)	47 (34%)	10 (40%)
> 30 min	27 (17%)	24 (17%)	3 (12%)
Prodromal symptoms (Past 3 months)	(*N* = 148)	(*N* = 125)	(*N* = 23)
Tingling	104 (70%)	85 (68%)	19 (83%)
Burning	75 (51%)	64 (51%)	11 (48%)
Itching	81 (55%)	67 (54%)	14 (61%)
Stinging	78 (53%)	64 (51%)	14 (61%)
Sensitivity to hot/cold	74 (50%)	59 (47%)	15 (65%)
Sensitivity to touch	79 (53%)	64 (51%)	15 (65%)
Pain	65 (40%)	55 (40%)	10 (40%)
Feelings of warmth	85 (57%)	69 (55%)	16 (70%)
Swelling	50 (34%)	42 (34%)	8 (35%)
Redness/discoloration	36 (24%)	27 (22%)	9 (39%)
Burst blood vessels (purple or dark spots)	8 (5%)	4 (3%)	4 (17%)
Blisters	12 (8%)	10 (8%)	2 (9%)
Scabbing	8 (5%)	7 (6%)	1 (4%)

### 4.2 Prodromal Symptoms

Approximately half of the individuals within each group (48% of those with liver disease and 49% of those without) reported that prodromal symptoms occur within 10 min of sunlight exposure. Only 12% of those with liver disease and 17% of those without liver disease indicated that they could be in sunlight for at least 30 min without experiencing prodromal symptoms (Table 2).

Several prodromal symptoms were more common among those with liver disease compared to those without liver disease, including tingling (83% vs. 68%), feelings of warmth (70% vs. 55%), sensitivity to touch (65% vs. 51%) and hot/cold (65% vs. 47%), itching (61% vs. 54%), and stinging (61% vs. 51%) (Table 2).

### 4.3 Impacts of EPP

The percentage reporting at least 1 phototoxic reaction in the previous 12 months was 60% for those with liver disease and 68% for those without liver disease. When asked about the extent to which their most recent phototoxic reaction affected their ability to do daily activities, 76% of those with liver disease reported “very much” compared with 55% of those without liver disease. Among those with liver disease, a larger percentage reported difficulty in doing chores (88%) and going for a walk, run, or bike ride (84%) for at least 15 min when it is sunny outside than those without liver disease (60% for both). A higher percentage of individuals with liver disease reported feeling depressed or sad (88% among those with liver disease vs. 73% of those without liver disease), anxious (92% vs. 78%), isolated (100% vs. 80%), frustrated (100% vs. 89%), and lonely (96% vs. 72%).

Fewer individuals with liver disease (44%) were working full time for pay than those without liver disease (55%), but those with liver disease reported missing more work due to EPP. Specifically, in the previous month, the mean (SD) number of hours missed from work for those reporting liver disease was much larger than for those not reporting liver disease (10.9 [24.5], compared to 3.6 [10.8]). Among the 21 individuals attending school (6 with liver disease and 15 without liver disease), the mean (SD) number of hours missed from school in the previous month was 7.7 (9.3) for those with liver disease and 3.6 (7.3) for those without liver disease.

The mean (SD) number of physician visits for EPP in the past 12 months was 16.5 (26.5) for those reporting liver disease and 6.0 (6.4) for those not. Participants in both groups were most likely to report that a general practitioner/internist is their primary physician for managing their EPP (25% for those reporting liver disease and 31% for those not reporting liver disease). The mean (SD) number of ER visits for EPP or complications was 9.0 (14.0) for those reporting liver disease and 1.9 (1.1) for those not reporting liver disease. A total of 7 individuals reporting liver disease also reported a hospitalization for EPP or complications, compared with 3 individuals without liver disease.

## 5 Discussion

The significant burden imposed by EPP appears to be greater among those with self‐reported liver disease. Compared to those without liver disease, individuals with liver disease experience a higher frequency of prodromal symptoms and report a greater negative impact of phototoxic reactions on their ability to do daily activities. Negative emotions associated with EPP are ubiquitous among those with liver disease: 100% reported feeling both isolated and frustrated, and over 90% reported being anxious and lonely (all higher percentages than among those without liver disease). The impact of EPP on work and school absenteeism also appears to be greater among those with liver disease: in the past month, the mean number of work hours missed was more than 3 times greater for those with liver disease, while the mean number of school hours missed was more than double. Individuals with liver disease also had more EPP‐related physician visits, ER visits, and hospitalizations in the previous 12 months than their counterparts without liver disease.

There are some significant limitations that should be noted. The sample size of those with liver disease, defined as having elevated liver enzymes or fibrosis, was obtained via self‐report and is relatively small (*n* = 25). The diagnosis was not confirmed by lab results or by imaging studies. In addition, although we asked whether participants had liver disease due to EPP, it is possible that they could have elevated liver enzymes due to other causes. Other factors such as obesity, high intake of alcohol, or diabetes could also cause elevated liver enzymes in someone with EPP. These other factors could contribute to difficulties with functioning. Finally, the analysis was descriptive in nature, and therefore no formal hypothesis testing was conducted.

EPP is known to have a negative impact on physical functioning and psychosocial well‐being [9, 14, 15]. The additional burden experienced by those with liver disease highlights the importance of early and continuing surveillance of liver function among those with EPP. In addition, those who develop liver disease should be evaluated to ensure they receive the necessary psychosocial support.

## Author Contributions

All authors made a significant contribution to the work reported, whether that is in the conception, study design, execution, acquisition of data, analysis and interpretation, or in all these areas; took part in drafting, revising, or critically reviewing the article; gave final approval of the version to be published; have agreed on the journal to which the article has been submitted; and agree to be accountable for all aspects of the work. S.D.M., H.H.C., and H.N. were primarily responsible for the design, content, and interpretation of the study.

## Ethics Statement

Human participants' research approval for the study was provided by an independent scientific review committee, WCG IRB.

## Consent

Informed consent was obtained from all participants.

## Conflicts of Interest

C.N., M.C., and W.S. are employed by and own stock and stock options in Disc Medicine. S.D.M. is an employee of Health Outcomes Solutions (HOS), and H.H.C. is a consultant to HOS., which received funding from Disc Medicine for the conduct of this study. K.W. has received research funding from Disc Medicine. H.N. consults for Alnylam Pharmaceuticals, Recordati Rare Diseases, Disc Medicine, and Mitsubishi Tanabe. M.B. has received honoraria for consulting from Alnylam Pharmaceuticals and Disc Medicine and funding for clinical trials from Alnylam Pharmaceuticals, Disc Medicine, and Mitsubishi Tanabe. The authors report no other conflicts of interest in this work.

## Data Availability

The datasets used and/or analyzed during the current study are available from the corresponding author upon reasonable request.
